# Supplementary material for: 3D Occlusal Tooth Wear Assessment in Presence of Limited Changes in Non-Occlusal Surfaces
Source: Diagnostics (Basel). 2021 Jun 4;11(6):1033. doi: 10.3390/diagnostics11061033 (PMC8228780; doi:10.3390/diagnostics11061033)
Supplement: Supplementary file 1 [file diagnostics-11-01033-s001.zip › 6.4/diagnostics-1236128-supplementary_NEW_20210604.docx]

**Supplementary Materials**

**3D occlusal tooth wear assessment in presence of limited changes in non-occlusal surfaces**

Nikolaos Gkantidis, Konstantinos Dritsas, Christos Katsaros, Demetrios Halazonetis and Yijin Ren


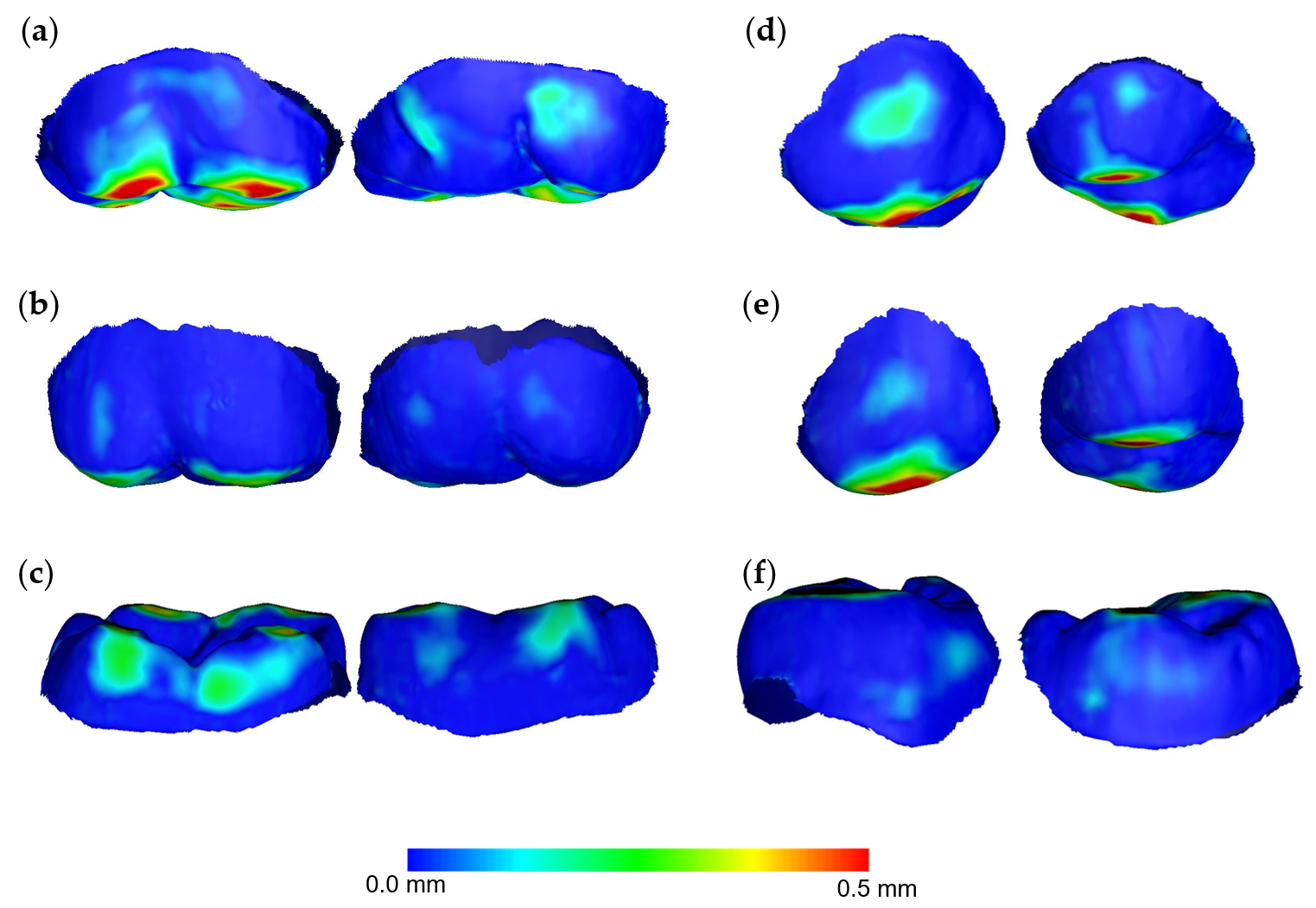


**Supplementary Figure S1.** Color coded distance maps showing the buccal and lingual aspects of superimposed tooth crowns, prior and after tooth wear simulation, using the gold standard t`echnique. Patterns of tooth wear simulation are shown from the buccal (left) and the lingual (right) aspects of: (**a**, **b**) two maxillary molars, (**c**) one mandibular molar, (**d**, **e**) two maxillary premolars, and (**f**) one mandibular premolar. The teeth shown were randomly selected from the total sample.


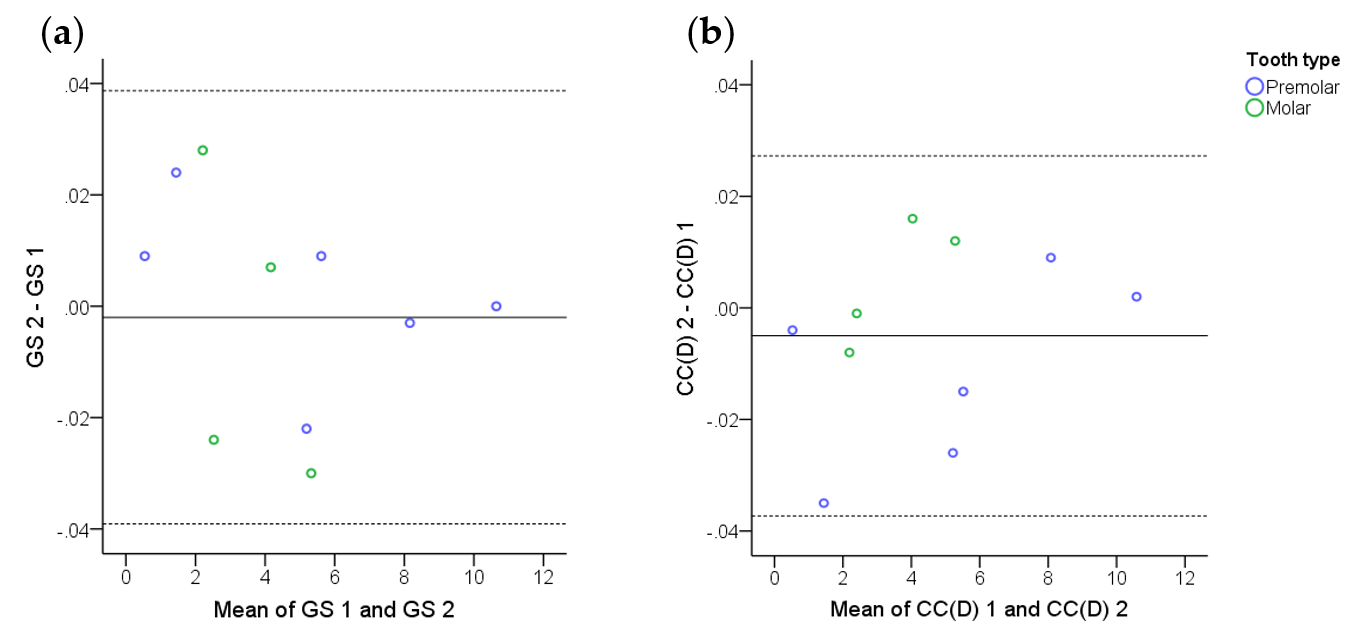


**Supplementary Figure S2.** Intra-operator error on tooth wear measurement (mm^3^) assessed through Bland Altman plots. (**a**) Gold standard (GS) technique. (**b**) Technique of choice (complete crown with 30% estimated overlap). The true range of measured tooth wear values is represented in the axes length. The continuous horizontal line shows the mean and the dashed lines the 95% confidence intervals.


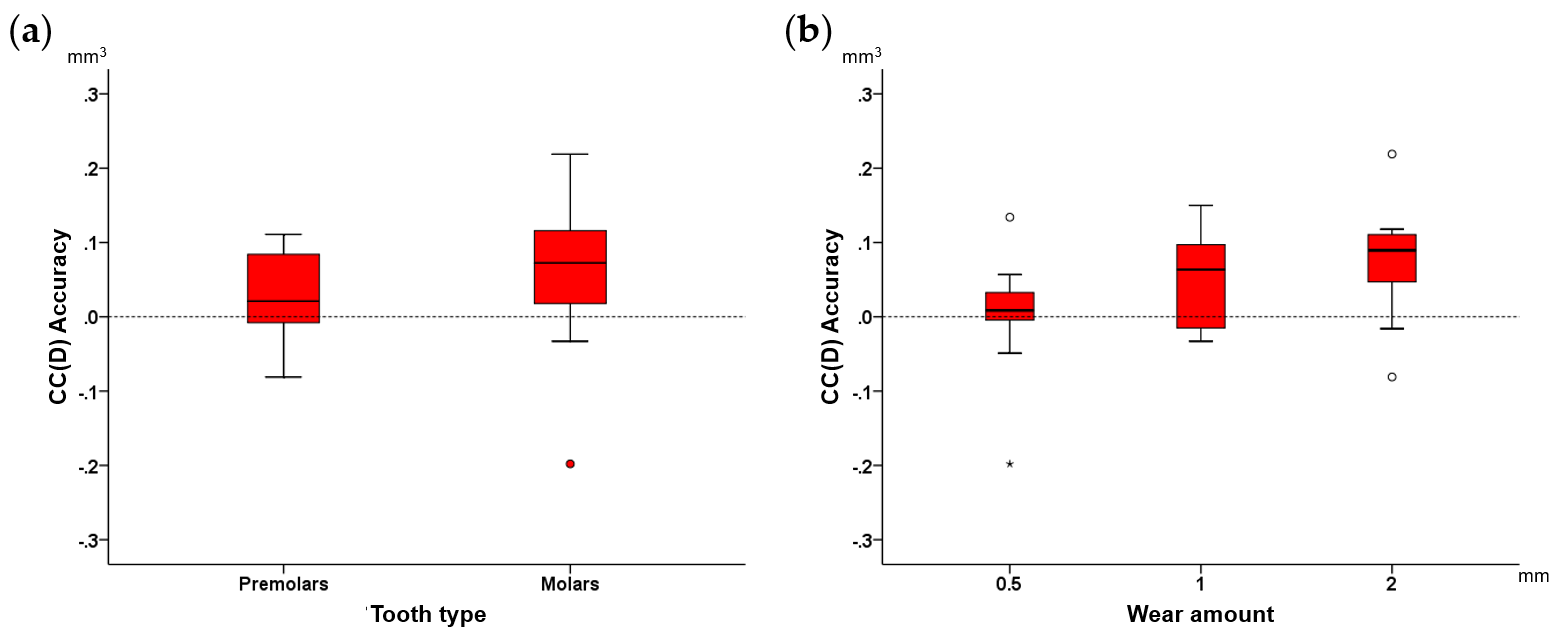


**Supplementary Figure S3.** Difference in tooth wear measurement between the technique of choice (complete crown, setting D) and the gold standard technique by (**a**) tooth type and (**b**) tooth wear amount. In the box plots, the upper limit of the black line represents the maximum value, the lower limit the minimum value, the box the interquartile range, and the horizontal black line the median value (trueness). Perfect agreement with the gold standard is indicated by zero value (horizontal dashed line). The vertical length of each plot indicates precision.


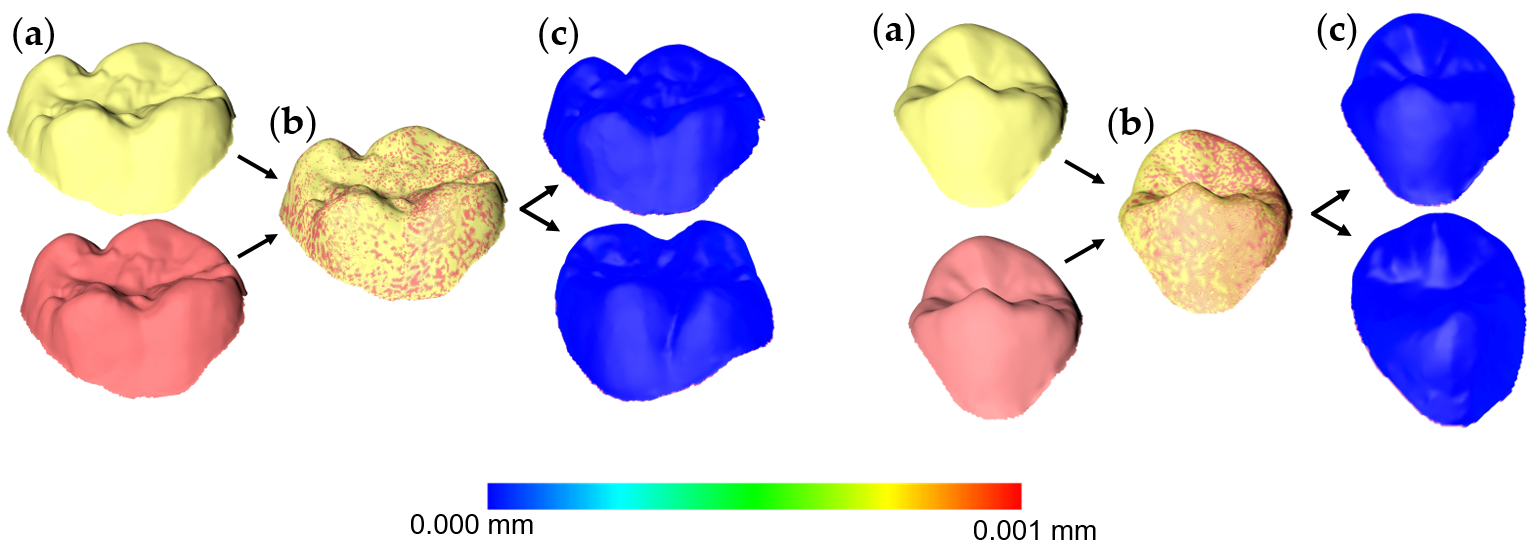
**Supplementary Figure S4.** Superimposition of identical surface models of a maxillary molar (left) and a maxillary premolar (right). (**a**) Original tooth (yellow) and duplicate (red). (**b**) Superimposed tooth crowns using the complete crown technique and setting D (30% estimated overlap) shown from the buccal aspect. (**c**) Color coded distance maps of the superimposed crowns, shown from the buccal (above) and the palatal (below) aspect.
